# Safety and utility of Endoscopic Ultrasound with Bronchoscope-guided Fine Needle Aspiration (EUS-B-FNA) in suspected lung cancer patients with poor respiratory or general conditions: a prospective three-center observational study

**DOI:** 10.1186/s12890-023-02508-2

**Published:** 2023-06-14

**Authors:** Koki Nakashima, Yukihiro Umeda, Yoshiki Demura, Toshihiro Takeda, Toshihiko Tada, Masayuki Sato, Norihiro Jikuya, Kosuke Kurokawa, Tomoaki Sonoda, Makiko Yamaguchi, Miho Mitsui, Masahiro Oi, Ryo Chikazawa, Yuko Waseda, Masaki Anzai, Masaya Akai, Tamotsu Ishizuka

**Affiliations:** 1 Third Department of Internal Medicine, Faculty of Medical Sciences, University of Fukui, 23-3 Matsuoka-Shimoaizuki, Eiheiji, Fukui 910-1193 Japan; 2 Department of Respiratory Medicine, Municipal Tsuruga Hospital, Fukui, Japan; 3 Department of Respiratory Medicine, Japanese Red Cross Fukui Hospital, Fukui, Japan

**Keywords:** Endoscopic ultrasound with bronchoscope-guided fine needle aspiration (EUS-B-FNA), Lung cancer, Driver oncogene, Programmed death ligand 1 (PD-L1), Next-generation sequencing (NGS)

## Abstract

**Background:**

Although transbronchial diagnostic procedures are sometimes difficult to perform because of the patient’s respiratory or general conditions, endoscopic ultrasound with bronchoscope-guided fine-needle aspiration (EUS-B-FNA), a known transesophageal diagnostic procedure, might be useful for such cases. We conducted this prospective three-center observational study to evaluate the safety and efficacy of EUS-B-FNA in suspected lung cancer patients with poor respiratory or general conditions.

**Methods:**

Patients with suspected lung cancer with respiratory failure, Eastern Cooperative Oncology Group performance status of 2 or higher, or severe respiratory symptoms, were enrolled. The primary endpoints were the diagnostic yield of lung cancer and its safety, and the secondary endpoints were the success rate of molecular and programmed death ligand 1 (PD-L1) analyses, and the 6-month survival rate in patients with lung cancer.

**Results:**

We enrolled 30 patients, of which 29 were included in the analysis. Among them, 26 were eventually diagnosed with lung cancer. The diagnostic yield for lung cancer was 100% (26/26). There were no adverse events associated with EUS-B-FNA requiring procedure discontinuation. The success rates of molecular analysis for *EGFR*, *ALK*, *ROS-1*, and *BRAF* were 100% (14/14), 100% (11/11), 100% (9/9), and 75% (6/8), respectively. The success rate of the PD-L1 analysis was 100% (15/15). The 6-month survival rate in patients with lung cancer was 53.8% (95% confidence interval [CI]: 33.4–76.4), and the median overall survival (OS) was 196 days (95% CI: 142–446).

**Conclusions:**

EUS-B-FNA is a safe and effective diagnostic method, even in patients with suspected lung cancer with poor respiratory or general conditions.

**Trial registration:**

This clinical trial was registered at https://www.umin.ac.jp/ctr/index.htm (UMIN000041235, approved on 28/07/2020).

## Background

Bronchoscopic procedures are widely used to diagnose lung cancer. Endobronchial ultrasound-guided transbronchial needle aspiration (EBUS-TBNA) has been reported as a safe and useful modality to diagnose lung cancer [1, 2]. Although transbronchial diagnostic procedures, including EBUS-TBNA, are minimally invasive, obtaining tumor tissues through the airway is difficult in patients with poor respiratory or general conditions in clinical practice. About 10–40% of patients with lung cancer are diagnosed following emergency admissions due to their symptoms [3–5], but little is known about safe diagnostic procedures in such cases.

On the other hand, the number of studies showing the efficacy of chemotherapies for patients with lung cancer with poor respiratory or general conditions is increasing in recent years. For example, molecularly-targeted drugs, such as epidermal growth factor receptor (*EGFR*)-tyrosine kinase inhibitor (TKI) or anaplastic lymphoma kinase (*ALK*)-TKI, are effective in improving performance status (PS) or respiratory conditions in driver oncogenes positive non-small cell lung cancer (NSCLC) patients even with poor PS [6, 7], or respiratory condition [8]. In addition, several studies [9, 10] suggested that immune checkpoint inhibitors (ICIs) have some clinical effectiveness in NSCLC patients with high programmed death ligand 1 (PD-L1) expression with poor PS. Fujimoto et al. [3] reported that appropriate chemotherapy and improvements of PS were associated with longer overall survival (OS) even in patients with lung cancer with poor respiratory or general conditions who were diagnosed following emergency admissions due to their symptoms. These findings highlight the importance of pathological and molecular diagnosis to determine appropriate treatments even for patients with suspected lung cancer with poor respiratory or general conditions. Therefore, there is a need for information on safe diagnostic methods that can obtain sufficient tumor samples for pathological and molecular diagnosis, even in such patients.

Endoscopic ultrasound with bronchoscope-guided fine needle aspiration (EUS-B-FNA) is a transesophageal diagnostic procedure to obtain tumor tissues adjacent to the esophagus. Both EUS-B-FNA and EBUS-TBNA are recommended for pathological or staging diagnosis of lung cancer in several guidelines [11, 12]. One advantage of EUS-B-FNA over EBUS-TBNA is that the former is a transesophageal procedure, which may be easier to perform than transbronchial procedures, regardless of respiratory conditions. Therefore, we hypothesized that EUS-B-FNA would be ideal for suspected lung cancer patients with poor respiratory or general conditions.

## Materials and methods

### Study design and patients

This was a multi-center, prospective observational study at University of Fukui Hospital, Japanese Red Cross Fukui Hospital, and Municipal Tsuruga Hospital, which was approved by the Ethics Committee of the Faculty of Medical Sciences, University of Fukui (Number 20200065, approved on 20/07/2020), the Institutional Review Board of Japanese Red Cross Fukui Hospital (Number R2-08–21, approved on 21/08/2020), and the Institutional Review Board of Municipal Tsuruga Hospital (Number 347, approved on 13/08/2020). This clinical trial was performed in accordance with the Declaration of Helsinki and registered at https://www.umin.ac.jp/ctr/index.htm (UMIN000041235, approved on 28/07/2020).

Eligibility criteria included age 20 years or older, and presence of tumor suspected to be lung cancer located adjacent to esophagus. The patients were also required to meet at least one following criteria: respiratory failure requiring supplemental oxygen to maintain oxygen saturation (SpO2) > 90%; Eastern Cooperative Oncology Group (ECOG) PS 2 or higher; or severe respiratory symptoms, such as cough with wheezing or stridor, that make transbronchial examinations unfeasible due to symptomatic lung cancer. Patients with esophageal varix, thrombotic disorders, or pregnancy were excluded. Moreover, we did not include patients with other safely biopsiable lesions, such as superficial lymph nodes, or whose respiratory status had improved with intervention, such as thoracic drainage, before EUS-B-FNA. All enrolled patients provided written informed consent for EUS-B-FNA and participation in this clinical study.

### EUS-B-FNA procedure

The procedure was performed as we have previously described [8]. EUS-B-FNA was performed after the patients had fasted for at least 4 h. In addition, local anesthesia with midazolam and analgesia with fentanyl or pentazocine could be used during EUS-B-FNA at the discretion of attending physicians.

Convex probe endobronchial ultrasonography (CP-EBUS; BF-UC260F or BF-UC290F [Olympus Corporation, Tokyo, Japan]) and 22-gauge EBUS-TBNA needles (NA-201SX-4022 or NA-U401SX-4022) were used in all cases. CP-EBUS was inserted through the esophagus. At least two tumor-punctures were required with real-time ultrasonographic guidance, except for cases with puncturing difficulties. The procedure time was defined as time from the first insertion to the exertion of the bronchoscope. Blood pressure, pulse rate, and SpO2 were monitored during the procedure. Supplemental oxygen was adjusted to maintain SpO2 > 90%, and the dosage of fluid replacement was adjusted to maintain a systolic blood pressure of 90 mmHg. Vital signs were monitored at least until the next morning after the procedure. All procedures were performed by pulmonologists at each institute. Rapid on-site cytologic examination (ROSE) was not performed.

Administration of prophylactic antibiotics and timing of oral intake resumption was decided at discretion of the attending physicians. Antithrombotic drugs were required to be rested, based on standard guideline [13].

The attending physicians examined the patients one week after EUS-B-FNA to confirm any adverse events after the procedure.

### Pathological diagnosis, molecular analysis, and PD-L1 analysis

Pathological diagnosis was made by pathologists at each institute. In patients with NSCLC, molecular and PD-L1 analyses were applied at the discretion of the attending physician, considering the amount of tumor specimen collected and turnaround time (TAT). Especially in molecular analysis, the attending physician also decided which analysis technique to use, such as singleplex test or next-generation sequencing (NGS), and which driver oncogenes to test for.

### Treatment and assessment

Treatment decisions and the choice of chemotherapy regimen were left to the attending physicians. Objective response rate (ORR) was evaluated according to Response Evaluation Criteria in Solid Tumor (RECIST) version 1.1 [14]. OS was defined as the time from the date of EUS-B-FNA to the date of death from any cause.

To assess the 6-month survival rate and late-onset adverse events, we followed the patients up for at least 6 months or until death.

### Outcomes

The primary endpoints were the diagnostic yield of lung cancer and safety. Diagnostic yield was defined as the rate of cases with pathologically diagnosed lung cancer with EUS-B-FNA. Severe adverse events were defined as requiring the extension of the hospitalization period, persistent dysfunction, life-threatening, or death. Any adverse events associated with EUS-B-FNA were recorded.

The secondary endpoints were the success rates of molecular analysis and PD-L1 analysis in patients with NSCLC who underwent these analysis, and the 6-month survival rate of patients diagnosed with lung cancer using EUS-B-FNA.

### Statistical analysis

OS was estimated using the Kaplan–Meier method, and 95% confidence intervals (CIs) were calculated. The date of data cut off was August 31st, 2022. Responses were summarized using frequency counts and percentages, and 95% CIs were calculated. Probability values < 0.05 were considered statistically significant. Statistical analyses were performed using EZR statistical software, version 1.55 (Y. Kanda, 2021).

## Results

### Patient characteristics

**Fig. 1:**
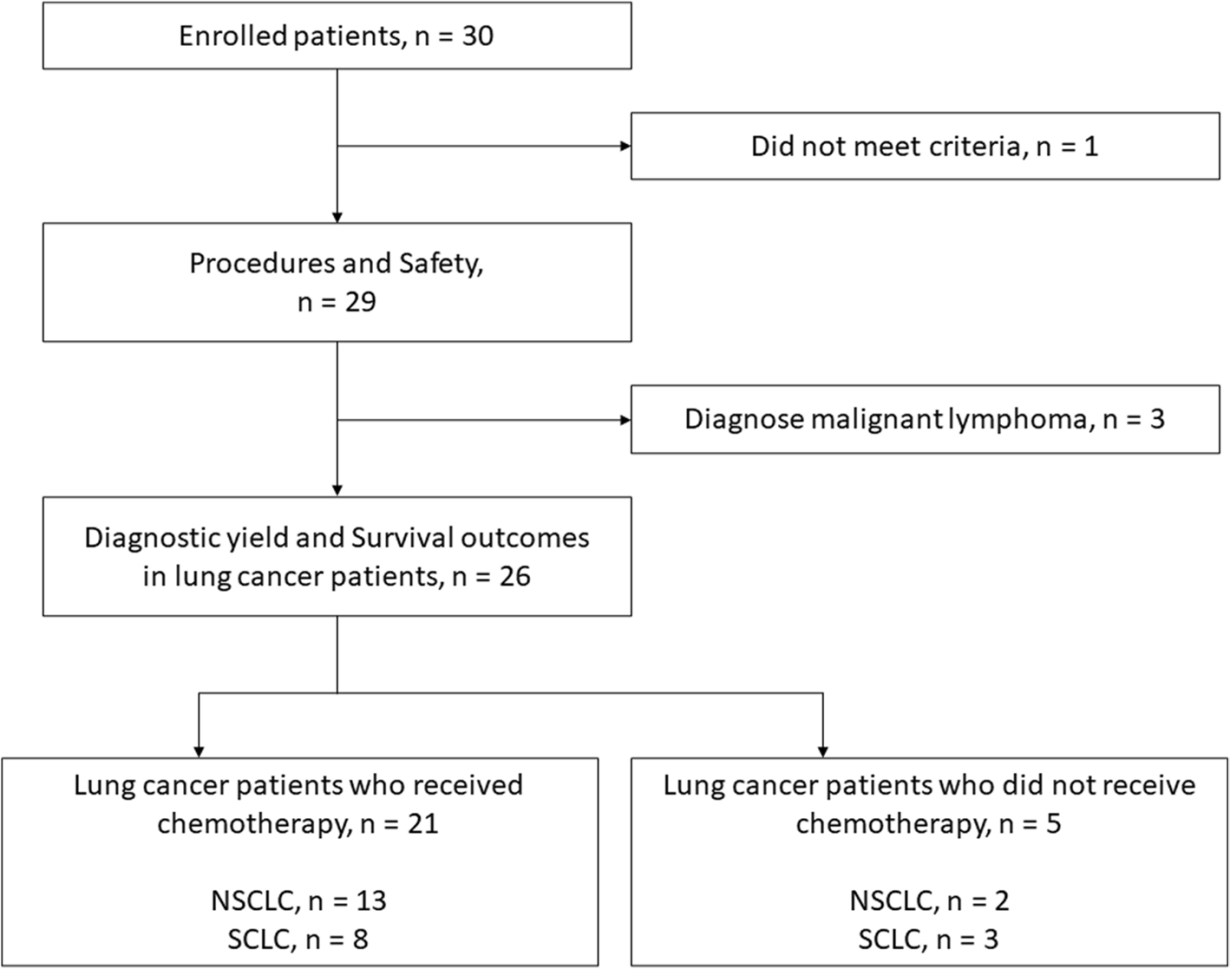
Flow diagram for patient selection

Between August 2020 and February 2022, 30 patients were enrolled in this study. One patient was excluded based on the eligibility criteria. This patient did not have a poor respiratory or general condition but was enrolled because a bronchoscopy was initially performed but interrupted due to poor sedation. Therefore, total 29 patients were analyzed. Since 3 patients were eventually diagnosed with malignant lymphoma, 26 patients with lung cancer were assessed for diagnosis and survival outcomes (Fig. 1).

Patient characteristics are shown in Table 1. The median age was 73 years (range 59–89), and 23 patients (79%) were male. Also, 24 patients (83%) were PS 2 or higher, and 18 (62%) were provided with supplemental oxygen at baseline. The main reasons for EUS-B-FNA were as follows: 18 patients (62%) with respiratory failure, 8 (28%) with poor PS, and 3 (10%) with severe respiratory symptoms (severe cough with stridor due to central airway obstruction caused by tumor). Final diagnosis was as follows: small cell lung cancer (SCLC) (*n* = 11), adenocarcinoma (*n* = 6), squamous cell carcinoma (*n* = 6), adeno-squamous carcinoma (*n* = 1), large cell neuroendocrine carcinoma (*n* = 1), NSCLC, not otherwise specified (NOS) (*n* = 1), and malignant lymphoma (*n* = 3).

**Table 1:** Patient characteristics

	*N* = 29 (%)
Age (years)	
Median	73
Range	59–89
Sex	
Male	23 (79)
Female	6 (21)
Smoking history	
Current smoker	12 (41)
Former smoker	15 (52)
Never smoked	2 (7)
Comorbidities	
Chronic respiratory disease	
Chronic obstructive pulmonary disease	13
Bronchial asthma	2
Tubeculosis sequela	1
Cardiovascular disease	
Coronary artery disease	3
Congestive heart failure	3
Cerebrovascular disease	3
Chronic Kidney disease	4
Others	
Hypertension	9
Diabetes melitus	7
Hyperthyroidism	2
Cervical cord injury	1
ECOG PS	
0–1	5 (17)
2	11 (38)
3	10 (35)
4	3 (10)
Supplemental oxygen (L/min)	
0	11 (38)
1	4 (14)
2	8 (28)
3	3 (10)
4-	3 (10)
Main reason for EUS-B-FNA	
Respiratory failure	18 (62)
Poor PS	8 (28)
Severe respiratory symptoms	3 (10)
Final diagnosis	
Lung cancer	26
Small cell lung cancer	11
Adenocarcinoma	6
Squamous cell carcinoma	6
Adenosquamous carcinoma	1
Large cell neuroendocrine carcinoma	1
Not otherwise specified	1
Other diseases	3
Malignant lymphoma	3
Stage of lung cancer	*N* = 26
IIIA	1
IIIB	2
IVA	11
IVB	11
Postoperative recurrence	1

*ECOG* eastern cooperative oncology group, *PS* performance status

### Procedures and safety

Details of the procedures and adverse events are shown in Table 2. The median total dose of midazolam was 4 mg (range 0–6.5). The median procedure time was 15 min (range 8–32). Among punctured lesions, 27 were mediastinal lymph nodes of #2L, #4L, #7, and #8, and 2 were intrapulmonary lesions. The median size of punctured lesions was 33 mm (range 17.5–77) in the major axis and 19.5 mm (range 11.1–39.1) in the minor axis. The median number of punctures was 3 (range 2–5). All patients received prophylactic antibiotics after EUS-B-FNA: intravenous ceftriaxone in 22 cases, intravenous levofloxacin in 2 cases, oral levofloxacin in 2 cases, intravenous ampicillin/sulbactam in 2 cases, and intravenous piperacillin/tazobactam in 1 case. For adverse events associated with EUS-B-FNA, oxygen desaturation to less than 90% occurred in 1 patient (3.4%), and oxygen desaturation to not less than 90% but requiring increased supplemental oxygen occurred in 14 patients (48.3%). However, the procedures could be continued after slightly increasing the supplemental oxygen dosage in all cases. Blood pressure reduction to less than 90 mmHg occurred in 4 patients (13.8%), but the procedure could be continued after raising the leg or increasing the dosage of fluid replacement. There was no bleeding requiring intervention, such as topical adrenaline or electrocoagulation. There were no adverse events requiring discontinuation of EUS-B-FNA nor severe adverse events.

**Table 2:** Details of procedures and adverse events

	*N* = 29
Midazolam (mg)	
Median	4
Range	0-6.5
Procedures time (minutes)	
Median	15
Range	8-32
Punctured lesion	
#2L	1
#4L	5
#7	20
#8	1
Intrapulmonary lesion	2
Punctured lesion size (mm)	
Major axis	
Median	33
Range	17.5-77
Minar axis	
Median	19.5
Range	11.1-39.1
Number of punctures	
Median	3
Range	2-5
Prophylactic antibiotics use	
Yes	29
No	0
Adverse events during procedure	
Oxygen desaturation to less than 90%	1
Oxygen desaturation to not less than 90% but requiring increased supplemental oxygen	14
Blood pressure reduction to less than 90 mmHg	4
Bleeding requiring intervention such as topical adrenaline or electrocoagulation	0
Adverse events requiring discontnuation of EUS-B-FNA	0
Adverse events after procedure	0
Severe adverse events during procedure	0
Severe adverse events after procedure	0

### Diagnostic yield, molecular analysis, and PD-L1 analysis

Diagnostic results are shown in Table 3. Among the 29 patients, 1 with malignant lymphoma could not be diagnosed using EUS-B-FNA; therefore, the diagnostic yield was 96.6% (28/29). Among patients with lung cancer, the diagnostic yield was 100% (26/26). One patient who could not be diagnosed with malignant lymphoma was eventually diagnosed with secondary EUS-B-FNA.

**Table 3:** Results of diagnosis

Diagnostic yield in all cases (%)	96.6 (28/29)
Diagnostic yield in lung cancer (%)	100 (26/26)
Success rates of molecular analysis in NSCLC (%)	
Singleplex for *EGFR*	100 (14/14)
Positive/Negative	1/13
Singleplex for *ALK*	100 (11/11)
Positive/Negative	0/11
Singleplex for *ROS-1*	100 (9/9)
Positive/Negative	0/9
Singleplex for *BRAF*	75 (6/8)
Positive/Negative	0/6
Success rate of PD-L1 analysis in NSCLC (%)	100 (15/15)
< 1%/1–49%/50%≦	7/4/4
Success rates of NGS analysis in lung cancer (%)	
Oncomine Dx Target Test	100 (4/4)
FoundationOne CDx	100 (1/1)

*NSCLC* Non-small cell lung cancer, *EGFR* Epidermal growth factor receptor, *ALK* Anaplastic lymphoma kinase, *ROS-1* c-ros oncogene 1, *BRAF* v-raf murine sarcoma viral oncogene homolog B1, *PD-L1* programmed death ligand 1, *NGS* next-generation sequencing

Of the 15 patients with NSCLC, 14, 11, 9, and 6 were tested singleplex for *EGFR* mutation, *ALK* fusion gene, c-ros oncogene 1 (*ROS-1*) fusion gene, and v-raf murine sarcoma viral oncogene homolog B1 (*BRAF*) mutation, respectively. The success rates of molecular analysis of these driver oncogenes were 100% (14/14), 100% (11/11), 100% (9/9), and 75% (6/8), respectively. *EGFR* mutation was detected in 1 patient. NGS with the Oncomine Dx Target Test was performed in 4 patients with NSCLC, and with FoundationOne CDx in 1 patient with SCLC. There was no case of failure to NGS analysis, but no treatable genetic mutations were found. One patient with stage IIIA NSCLC was tested using NGS. Therefore, all patients with NSCLC were evaluated as having *EGFR* mutation.

The PD-L1 tumor proportion score was successfully analyzed in all patients with NSCLC.

### Survival outcomes and treatment benefits in patients with lung cancer

**Fig. 2:**
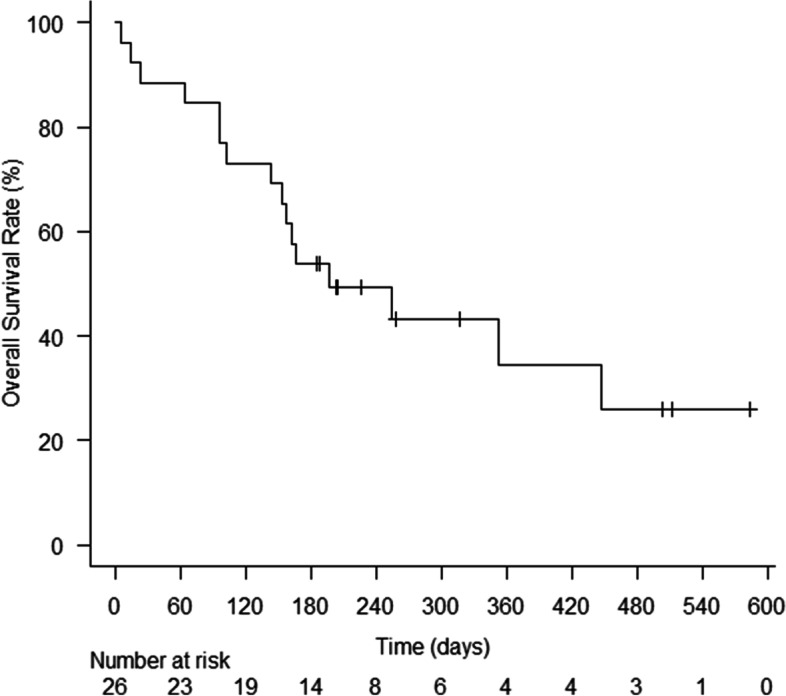
Kaplan–Meier estimates for overall survival in all patients with lung cancer. The median overall survival (OS) was 196 days (95% CI: 142-not reached [NR])

Among the 26 patients with lung cancer, the 6-month survival rate was 53.8% (95% CI: 33.4–76.4), and the median OS was 196 days [95% CI: 142-not reached (NR)] (Fig. 2). In total, 21 patients received chemotherapy (13 patients with NSCLC and 8 patients with SCLC). Among the 13 patients with NSCLC who received chemotherapy, 5 received platinum-doublet + ICI(s), 3 received platinum-doublet, 4 received cytotoxic monotherapy, and 1 received *EGFR*-TKI. Among the 8 patients with SCLC who received chemotherapy, 5 received platinum-doublet + ICI, 2 received platinum-doublet, and 1 received cytotoxic monotherapy. One patient achieved complete response, 13 had partial response, 3 had stable disease, and 4 had progressive disease. The ORR was 66.7% (95% CI: 43–85.4), and the disease control rate was 81% (95% CI: 58.1–94.6).

Two patients diagnosed with malignant lymphoma via EUS-B-FNA also had favorable progress with chemotherapy.

## Discussion

This prospective observational study evaluated the safety and efficacy of EUS-B-FNA in patients with suspected lung cancer with poor respiratory or general conditions. EUS-B-FNA was a highly safe diagnostic procedure for such patients and had a high diagnostic yield and high success rates of molecular and PD-L1 analysis. Therefore, EUS-B-FNA can be a useful diagnostic option in cases where no other site can be safely biopsied or where therapeutic interventions, such as thoracic drainage, are not expected to improve respiratory or general conditions.

Similar to our results, a recent meta-analysis of EUS-B-FNA, reviewing 10 studies, also reported no serious adverse events associated with EUS-B-FNA [15], although there were no studies limited to patients with poor respiratory or general conditions. Another meta-analysis of EUS-FNA reported mediastinitis, esophageal perforation, and mediastinal bleeding as severe adverse events of EUS-FNA. However, it concluded that this procedure was safe because the frequency of severe adverse events was very rare (0.3%) [16]. Our study also demonstrates the safety of EUS-B-FNA in patients with poor respiratory or general conditions, which may be due to the following reasons. First, because EUS-B-FNA is a transesophageal procedure, it can be performed with fewer changes in respiratory conditions or symptoms than transbronchial procedures. There was no discontinuation of procedures due to worsening respiratory conditions or symptoms in our study, although controllable fluctuations in vital signs occurred in some cases. Second, EUS-B-FNA can be performed with a relatively small dose of sedative. The median total dose of midazolam was 4 mg (range 0–6.5) in our study; it could be safely administered. Third, the EUS-B-FNA procedure time was short (median: 15 min). In a randomized study comparing EUS-B-FNA to EBUS-TBNA for the diagnosis of mediastinal lesions, Oki et al. [17] also reported that EUS-B-FNA had fewer cases of oxygen desaturations and cough during the procedure, smaller doses of a sedative drug, and shorter procedure time than EBUS-TBNA. These factors may make EUS-B-FNA more tolerable for patients with poor respiratory or general conditions.

It is unclear whether prophylactic antibiotics should be administered after EUS-B-FNA. In a meta-analysis of EUS-FNA [16], Bartheld et al. reported that cystic lesions or sarcoidosis might have some risk of increasing mediastinal infections. All patients in our study received prophylactic antibiotics after EUS-B-FNA, and there were no infectious adverse events. Therefore, prophylactic antibiotics may have some benefits even for patients with suspected lung cancer.

The efficacy of EUS-B-FNA in patients with suspected lung cancer with poor respiratory or general conditions was sufficient, with a diagnostic yield of 96.6% in all enrolled patients and 100% in lung cancer patients. Notably, among the 26 patients diagnosed with lung cancer using EUS-B-FNA, the rate of SCLC (42.3%) was higher compared to the general epidemiology, which presumably resulted from the large number of patients having a smoking history and poor respiratory conditions, which may have led to more central-type lung cancers. Although at least two punctures were required, the condition was achieved in all cases, and the median number of punctures was three. In a clinical study evaluating the optimal number of punctures with EBUS-TBNA in the mediastinal staging of NSCLC in the absence of ROSE, Lee et al. [18] recommended at least two punctures. Multiple punctures with EUS-B-FNA are easy to perform due to the softness of the esophageal wall, which may have a positive effect on diagnosis.

We also demonstrated high success rates of molecular and PD-L1 analysis with tumor samples obtained using EUS-B-FNA. Molecular and PD-L1 analysis, as well as pathological diagnosis, are extremely important to determine appropriate treatments for lung cancer. Although surgical biopsies can obtain larger tumor samples and are reliable for molecular and PD-L1 analysis, it has been suggested that these analyses can also be performed using FNA samples. Several studies reported that tumor samples obtained with FNA, including EBUS-TBNA and EUS-B-FNA, had high success rates of molecular [19, 20], and PD-L1 analysis [21, 22]. In addition, Sakakibara et al. suggested EBUS-TBNA as a promising method for PD-L1 analysis, showing good concordance between EBUS-TBNA samples and surgical resected samples, greater number and lower crash rates of tumor cells in EBUS-TBNA samples than in transbronchial biopsy (TBB) samples [23]. EUS-B-FNA can be a diagnostic option for molecular and PD-L1 analysis, as well as pathological diagnosis in patients with suspected lung cancer with poor respiratory or general conditions. It could be a useful tool for patient stratification.

Considering our experience, we believe that EUS-B-FNA has the following advantages over EUS-FNA in patients with poor respiratory or general conditions. First, EUS-B-FNA is performed by pulmonologists, whereas EUS-FNA is performed by gastroenterologists. In other words, EUS-B-FNA may be more suitable for patients requiring appropriate respiratory management. Moreover, the time to diagnosis may be shortened because consultation with gastroenterologists is not required. Second, the bronchoscope used in EUS-B-FNA is thinner in terms of outer diameter than the endoscope used in EUS-FNA, which may facilitate a less invasive procedure in patients with poor respiratory conditions. Furthermore, no inconveniences were associated with the using the bronchoscope for transesophageal examination. Therefore, EUS-B-FNA may be a technique that pulmonologists should master.

Despite the clinically relevant results, our study has some limitations. First, this study was conducted at a limited number of institutes with a small sample size. High diagnostic yield and safety may be difficult to generalize, as they may be due to the experience of the operators and diagnostic team. For example, all operators in this study had at least 2 years of training at bronchoscopy-training facilities, but it is unclear whether less experienced diagnostic teams could achieve similar results. Therefore, further large-scale multicenter studies are needed to address these challenges. However, our study has clinical value since collecting patients with poor respiratory or general conditions is relatively difficult. Second, the sample size was too small to assess the validity of samples obtained with EUS-B-FNA for molecular analysis and NGS. Moreover, molecular analysis was only a secondary endpoint that did not strictly prescribe testing methods, and only a limited number of types of driver oncogenes were tested with singleplex tests. For example, there were no cases in which mesenchymal-epithelial transition (*MET*) or Kirsten rat sarcoma viral oncogene homolog (*KRAS*) were tested with a singleplex test. Since this study enrolled patients with poor respiratory or general conditions, attending physicians tested only singleplex for *EGFR* and PD-L1 analysis but not other singleplex tests or NGS in most cases because of the importance of TAT. However, in cases tested with singleplex for other driver mutations or NGS, the success rates of molecular analysis and NGS were relatively high, and samples obtained with EUS-B-FNA may be suitable for these examinations. The softness of the esophageal wall may facilitate multiple punctures, which may allow obtaining a sufficient amount of tumor tissue. Further studies are needed to assess the validity of EUS-B-FNA samples for molecular analysis and NGS. Finally, it is difficult to assess whether chemotherapy according to pathological diagnosis should be provided to patients with poor respiratory or general conditions. Recently, molecularly-targeted drugs have proven effective even for patients with poor respiratory or general conditions [6–8]. Moreover, ICIs have also proven effective for such patients with high PD-L1 expression [9, 10]. Conversely, it remains unclear whether chemotherapy should be provided to such patients without driver oncogenes or with low PD-L1 expression. However, in our study, the patients who received chemotherapy had some clinical benefits with an ORR of 66.7%. Further studies are needed to evaluate the survival benefit for such patients, especially those without driver oncogenes or with low PD-L1 expression.

In conclusion, this is the first prospective study to evaluate the safety and efficacy of EUS-B-FNA in patients with suspected lung cancer with poor respiratory or general conditions. We have demonstrated that EUS-B-FNA is a safe and effective diagnostic method in such patients. EUS-B-FNA should be considered as a diagnostic option in patients with suspected lung cancer with poor respiratory or general conditions.

## Data Availability

All data generated or analyzed during this study are included in this article. Further inquiries can be directed to the corresponding author.

## Abbreviations

ALK: Anaplastic lymphoma kinase
BRAF: V-raf murine sarcoma viral oncogene homolog B1
CI: Confidence interval
EBUS-TBNA: Endobronchial ultrasound-guided transbronchial needle aspiration
ECOG: Eastern Cooperative Oncology Group
EGFR: Epidermal growth factor receptor
EUS-B-FNA: Endoscopic ultrasound with bronchoscope-guided fine-needle aspiration
ICI: Immune checkpoint inhibitor
NGS: Next generation sequencing
NOS: Not otherwise specified
NR: Not reached
NSCLC: Non-small cell lung cancer
ORR: Objective response rate
OS: Overall survival
PD-L1: Programmed death ligand 1
PS: Performance status
RECIST: Response Evaluation Criteria in Solid Tumor
ROSE: Rapid on-site cytologic examination
ROS-1: C-ros oncogene 1
SCLC: Small cell lung cancer
TBB: Transbronchial biopsy
TKI: Tyrosine kinase inhibitor
